# Influence of Type and Concentration of Biopolymer on β-Carotene Encapsulation Efficiency in Nanoemulsions Based on Linseed Oil

**DOI:** 10.3390/polym14214640

**Published:** 2022-10-31

**Authors:** Jenifer Santos, Luis A. Trujillo-Cayado, Marina Barquero, Nuria Calero

**Affiliations:** 1 Facultad de Ciencias de la Salud, Universidad Loyola Andalucía, Avda. de las Universidades s/n, 41704 Dos Hermanas, Sevilla, Spain; 2 Departamento de Ingeniería Química, Escuela Politécnica Superior, Universidad de Sevilla, c/Virgen de África 7, E41007 Sevilla, Spain; 3 Departamento de Ingeniería Química, Facultad de Química, Universidad de Sevilla, c/Profesor García González 1, E41012 Sevilla, Spain

**Keywords:** linseed oil, nanoemulsions, guar gum, Advanced Performance xanthan gum, β-carotene

## Abstract

Many lipophilic active substances, such as β-carotene, are sensitive to chemical oxidation. A strategy to protect these ingredients is encapsulation using nanoemulsions. This work analyzes the relationship between the physical stability and encapsulation efficiency of nanoemulsions based on linseed oil. The role of two different polysaccharides, Advanced Performance xanthan gum (APXG) or guar gum (GG) as stabilizers at different concentrations were studied to reach the required physical stability of these systems. This was investigated by means of droplet size distributions, steady-state flow curves, small amplitude oscillatory shear tests, multiple light scattering, and electronic microscopy. The overall results obtained reveal a depletion flocculation mechanism in all the APXG nanoemulsions, regardless of the concentration, and above 0.3 wt.% for GG nanoemulsions. Moreover, it has been demonstrated that enhanced physical stability is directly related to higher values of encapsulation efficiency. Thus, the nanoemulsion formulated with 0.2 wt.% GG, which presented the lowest creaming degree conditioned by depletion flocculation, showed a relative β-carotene concentration even above 80% at 21 days of aging time. In conclusion, the adequate selection of polysaccharide type and its concentration is a key point for the application of stable nanoemulsions as vehicles for active ingredients.

## 1. Introduction

Important changes have been taking place in consumer habits, for example, healthy eating, which is a growing trend in recent years. Numerous scientific pieces of evidence about the role of diet and its relationship with wellness and health have favored the appearance of functional foods, which is currently one of the main drivers of the development of new products [1]. Functional foods are based on the occurrence of functional ingredients (bioactive compounds) that exhibit beneficial effects for health. In this sense, the use of carotenoid family substances in food products, such as β-carotene, lycopene, lutein, and astaxanthin has aroused great interest for decades in the food industry for their potential health benefits due to their provitamin A activity and antioxidant properties. However, their applications are limited due to their insolubility in water and slight solubility in oil at room temperature [2] and their instability to heat, light, and air [3].

Among the technological strategies for the development of these functional foods, it is worth highlighting the use of oil-in-water (O/W) emulsions, which protect the active compound from chemical degradation and improve the water solubility and bioavailability of carotenoids dissolved within the oil phase of O/W emulsions [2,4,5]. Oil-in-water emulsions consist of a nonpolar oily phase that forms dispersed droplets in a polar aqueous phase. In general, emulsions are thermodynamically unstable, and the phases that compose them tend to separate by different mechanisms [6]. However, the emulsions can be kinetically stabilized, even for long periods of time, using two types of substances, emulsifiers and stabilizers, which delay or inhibit the destabilization processes. Emulsifiers are molecules with surface activity and therefore tend to adsorb on the surface of the droplets forming a protective barrier that prevents their aggregation. On the other hand, stabilizers are frequently used to slow down the destabilization process of emulsions and control their texture [7], increasing the viscosity of the continuous phase of the emulsion and promoting the formation of a network of oil droplets [8].

It is of paramount importance that to preserve the properties of these active substances using an emulsion-type format, this must possess the appropriate composition and stability. Previous studies have demonstrated that the composition of emulsions and the environment including emulsifiers, light, heat, etc. condition the degradation of carotenoids in emulsions [4,5]. Thus, many studies have analyzed the formation and stability evaluation of carotenoid emulsions. Ax et al. [9] investigated the influence of thermal treatment temperature and oxygen content on the degradation kinetics and isomerization of lycopene in O/W emulsions. Moreover, it has been demonstrated that the oxidative stability of carotenoids also depends on the type of oil used to form the emulsion [10]. In another study, the stability of protein-stabilized β-carotene nanodispersions has been evaluated in relation to heating, salts, and pH conditions [11]. Boon et al. have analyzed, in different studies, the influence of surfactant and oil types, pH, and the occurrence of antioxidants on the oxidation of lycopene contained in O/W emulsions [4,5]. Furthermore, the influence of processing variables on the chemical stability of β-carotene has been proved [12].

On the other hand, linseed oil, used in this work, is considered a functional oil because it can produce metabolic and physiological effects and health benefits in addition to providing basic nutritional functions. Regular consumption of functional oils can bring numerous benefits to the body, beauty, and health. A small daily dose of linseed oil may prevent heart disease and control cholesterol and blood pressure. Additionally, it possesses an anti-inflammatory action, and it helps the gastrointestinal system due to its anti-cancer and antioxidant compounds [13]. These properties are related to the composition of linseed oil, formed by 9% saturated fat, 18% monounsaturated fat, and 73% polyunsaturated fat (57% omega 3 and 16% omega 6) [14,15]. Moreover, it contains a high amount of soluble fiber, vitamins such as A, E, B1 or B2, iron, zinc, iron, and some potassium, magnesium, calcium, and phosphorous [16].

Appyclean 6552 is a non-ionic surfactant made up of amyl, capryl, and lauryl xylosides obtained from wheat. Its plant origin makes the use of this surfactant rather safe due to its rapid biodegradability and low toxicity to the environment (OECD 301F and OECD 311). Its formulation based on pentoses (monosaccharides), instead of glucose, makes it possible to react at lower processing temperatures and greater polymerization control. Compared to glucose-based surfactants, the degree of polymerization and its hydrophilic head are lower. This surfactant has an HLB value between 11.5 and 12 (Wheatoleo).

Vegetable gums such as xanthan gum and guar gum are used as thickeners and gelling agents in a multitude of food products. Both gums are accepted as safe food additives and they are rather useful in gluten-free recipes to achieve the typical texture and viscosity. While xanthan gum is produced by the bacterial fermentation of corn flour starches (*Cyamopsis tetragonoloba*) [17], guar gum is extracted from the seed endosperm of a leguminous plant [18]. Both polysaccharides are strongly different in molecular and structural terms, which leads to different rheological behavior and thickener properties [19].

The overall objective of this work is the use of two different types of polysaccharides, xanthan and guar gums as stabilizers and Appyclean 6552 as an emulsifier in linseed oil-based nanoemulsions developed for the encapsulation systems of beta-carotene. To be more precise, the specific goal of this project was to study the relationship between nanoemulsion physical stability and the encapsulation efficiency of beta-carotene in these systems.

## 2. Materials and Methods

### 2.1. Materials

The dispersed phase used was linseed oil, supplied by the local supermarket. β-Carotene, which was encapsulated in the dispersed phase, was supplied by Sigma Chemical Company. The continuous phase contained an eco-friendly surfactant, Appyclean 6552, as an emulsifier (Wheatoleo, Pomacle, France) and both gums (Advanced Performance xanthan gum and guar gum) were donated by CP Kelco. They were used as received and the gums solutions and nanoemulsions were prepared using deionized water.

### 2.2. Methods

#### 2.2.1. Preparation of Linseed Oil Nanoemulsions

The final amount of samples prepared was 250 g. The aqueous phase was formulated using deionized water and 3 wt.% of Appyclean 6552. The oil phase (30 wt.%) contained pure linseed oil and 0.1 wt.% rosemary essential oil as a natural preservative. First, a coarse emulsion was prepared using the Silverson L5M (Silverson, Chesham, UK). This rotor-stator was equipped with a mesh screen specially designed for the preparation of fine nanoemulsions. The samples were homogenized at 8000 rpm for 1 min. Secondly, the reduction of droplet size was carried out using a Microfluidizer (M-110P, Microfluidics, USA) at 15,000–25,000 psi. The configuration used in the Microfluidizer was Y + Z due to its advantages [20]. Water at 5 °C was used to refrigerate the outflow sample tube of the Microfluidizer to minimize the possible recoalescence.

Once the processing parameters are settled, the nanoemulsion was selected to incorporate the biopolymers: Advanced Performance xanthan gum (APXG) and guar gum (GG). Three samples of each were prepared using different concentrations (0.1, 0.2, and 0.3 wt.%). The stock solution for both gums was prepared by dissolving 1 wt.% powder in deionized water. The protocol described by Santos et al., 2020 [21] was followed to prepare the gums.

#### 2.2.2. Encapsulation of β-Carotene

An amount of 0.2 wt.% of β-carotene was dispersed into the dispersed phase, and then the mixture was sonicated and heated for 15 min at 40 °C to obtain the total dissolution. Subsequently, the same procedure described above was followed to form the nanoemulsions.

#### 2.2.3. Laser Diffraction Measurements

Malvern Mastersizer 2000 (Malvern, Worcestershire, UK) was used to characterize the droplet size distributions of the nanoemulsions developed. To quantify the mean droplet sizes and the polydispersity, different parameters were used: Sauter mean diameter (*D*_3,2_), volumetric diameter (*D*_4,3_), and span.

(1) $$D_{3,2}=\sum_{i=1}^{N}n_{i}d_{i}^{3}/\sum_{i=1}^{N}n_{i}d_{i}^{2}\;$$

(2) $$D_{4,3}=\sum_{i=1}^{N}n_{i}d_{i}^{4}/\sum_{i=1}^{N}n_{i}d_{i}^{3}\;$$

(3) $$span=\frac{D_{90}-D_{10}}{D_{50}}$$

where *d*_i_ is the droplet diameter, *N* is the total number of droplets, *n*_i_ is the number of droplets having a diameter *d*_i_, and *D*_90_, *D*_50_, and *D*_10_ are the diameters at 90%, 50%, and 10% cumulative volume, respectively.

#### 2.2.4. Rheological Characterization

A controlled-stress rheometer (Haake MARS II; Thermo Fisher Scientific, Waltham, MA, USA) equipped with a serrated plate–plate geometry (60 mm diameter) was used to study the flow and the oscillatory behaviors of the samples at 20 °C. On the one hand, a multi-step protocol was followed to characterize the flow curve (3 min/point). On the other hand, small amplitude oscillatory tests were carried out (20–0.05 rad/s) at a stress below the critical one. This critical stress was obtained by means of stress sweeps at 0.1, 1, and 3 Hz. All rheological tests were measured in triplicate.

#### 2.2.5. Study of Physical Stability

The physical stability of the systems developed was characterized and quantified by means of the multiple light scattering technique using Turbiscan Lab Expert (Formulaction, Toulouse, France). Backscattering measurements were conducted for a maximum of 20 days and a minimum of 1 day at 25 °C, depending on the stability of the sample.

#### 2.2.6. Cryo-Scanning Electron Microscopy

A cryo-scanning electron microscope Zeiss EVO (Zeiss, Oberkochen, Germany) was used to observe some selected nanoemulsions at 5–10 kV. Samples were prepared following the protocol reported by Santos et al. (2017) [22].

#### 2.2.7. β-Carotene Entrapment Efficiency and Degradation

The encapsulation efficiency of β-carotene in the nanoemulsions was determined by analyzing the concentration of the active ingredient in the samples using spectrophotometry. The protocol to prepare the samples was previously reported by Santos et al. [4]. The results for the β-carotene degradation were represented as relative concentrations of β-carotene: C/C_0_, C, and C_0_ are the concentrations of the compound at a specific time and just after preparation, respectively.

## 3. Results and Discussion

### 3.1. Influence of Processing Variables on Droplet Size Distribution for Linseed Oil Nanoemulsions

**Figure 1:**
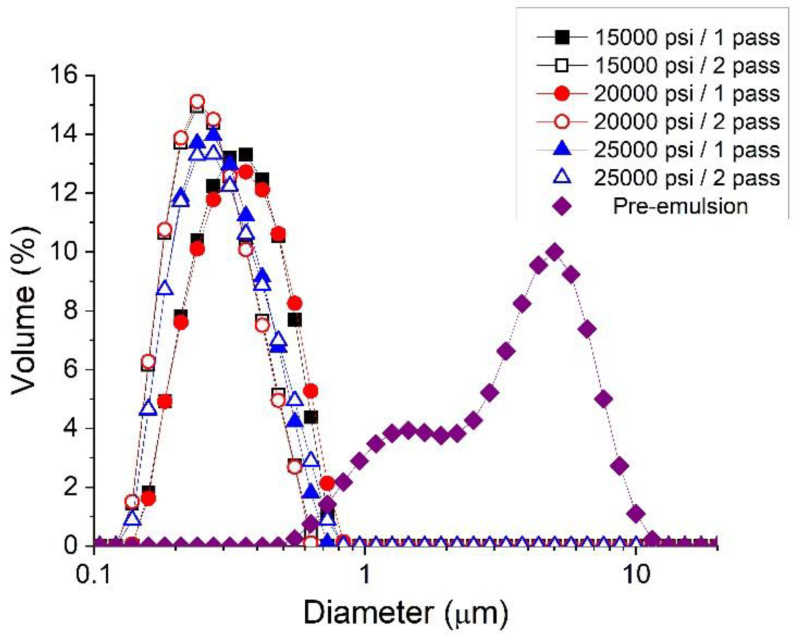
Droplet size distribution for nanoemulsions formulated with 40 wt.% of linseed oil as a function of homogenization pressure and number of passes in the Microfluidizer M110P.

Figure 1 shows droplet size distributions for emulsions containing linseed oil without gum as a function of processing variables used in the Microfluidizer. As expected, droplet size was reduced by using the microfluidization technique to reach the nanometric scale. The critical size to distinguish a nanoemulsion from a conventional emulsion is not clear. Some authors suggest the limit for nanoemulsions is 700 nm [23], 500 nm [24], 200 nm [25], and 100 nm [26]. There is a slight decrease in Sauter and volumetric diameters at the highest homogenization pressure (Table 1). In addition, this decrease is also shown at the lower homogenization pressures with two passes through the Microfluidizer. However, a decrease in mean diameter is not observed from one to two passes at 25,000 psi. This fact could suggest that the minimum mean diameter using this formulation is reached. On top of that, recoalescence, which is very common when using microfluidizers, did not take place, suggesting a protected interface thanks to the surfactant used. On the other hand, the span values obtained were around 1. This fact indicates that the polydispersity of droplets is high and therefore, there are droplets from 100 nm to 1 micron. The sample processed at 15,000 psi after two passes through the Microfluidizer showed the lowest mean diameters; hence, this nanoemulsion was chosen as a reference to incorporate different stabilizers.

### 3.2. Incorporation of Guar Gum and Advanced Performance Xanthan Gum in Linseed Oil Emulsions

**Figure 2:**
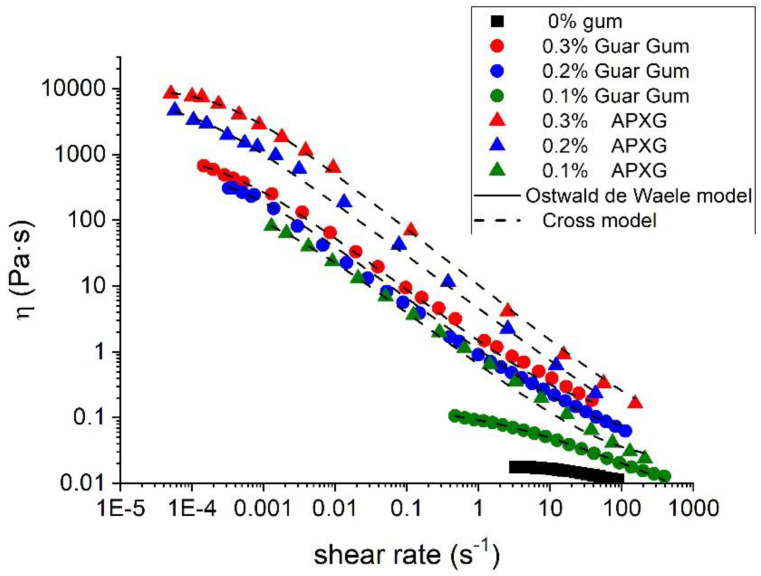
Flow behavior for linseed oil emulsions as a function of gum type and gum concentration.

Figure 2 shows the flow behaviors for the emulsions developed. Linseed oil emulsion without any gum showed a shear thinning behavior without a tendency to reach a plateau. These points fitted fairly well to the well-known Ostwald de Waele model. Interestingly, the incorporation of not only guar gum but also xanthan gum provoked an increase in shear thinning behavior and a tendency in viscosity values to reach a plateau at very low shear rates. Viscosity–shear rate values of emulsions containing gums were fitted to the Cross model (R^2^ > 0.99). This points out the increase in structuration grade due to the gum occurrence. Regardless of gum type, there is an increase in zero-shear viscosity with gum concentration. This fact is totally expected since polysaccharides have been used as thickeners for many years [27,28,29]. Concerning guar gum incorporation, a decrease in flow index is observed from 0.1 wt.% to 0.2 wt.% and then, it was constant above 0.2 wt.% GG. This fact indicates an increase in structuration grade from 0.1 wt.% to 0.2 wt.%. Concerning Advanced Performance xanthan gum (APXG) incorporation, the flow index seems to have a slight tendency to decrease with gum concentration. However, these differences are not significant (ANOVA test). The values of zero-shear viscosity of emulsions formulated with APXG were higher than the counterparts formulated with guar gum. This fact suggests the strong entanglement and the large network of APXG emulsions or highly flocculated emulsions due to the incorporation of APXG, compared with GG emulsions [30]. Furthermore, there is a big increase in viscosity values from 0 to 0.1 GG/APXG and from 0.1 GG/APXG to 0.2 GG/APXG. However, the values of viscosities (see Table 2) seem to tend to stabilize from 0.2 to 0.3 wt.% gum, regardless of the gum type.

**Figure 3:**
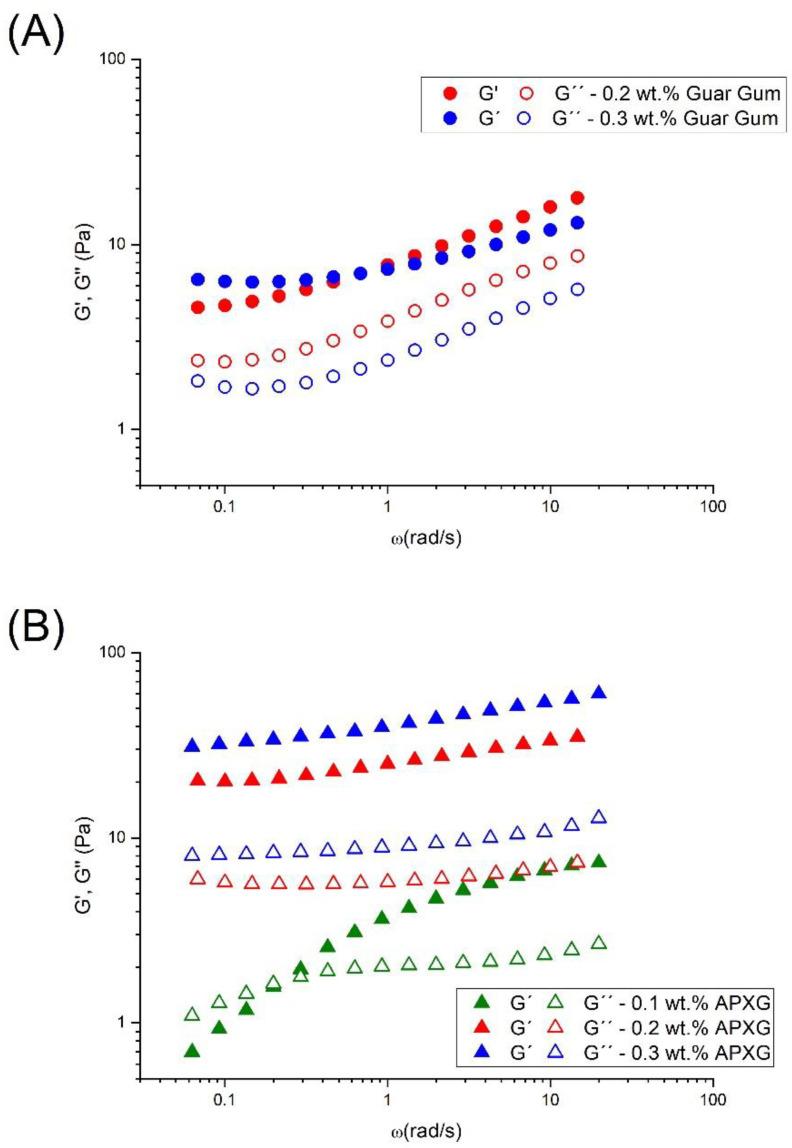
Frequency sweeps for linseed oil emulsions as a function of gum concentration for systems formulated with (**A**) guar gum and (**B**) Advanced Performance xanthan gum.

Figure 3A,B illustrate the mechanical spectra for emulsions containing linseed oil as a function of gum concentration for GG emulsions and APXG emulsions, respectively. It is important to note that the emulsion prepared without both gums and with 0.1 wt.% GG does not present significant viscoelastic properties at the lowest stress allowed by the rheometer since its colloidal structure was probably destroyed by the lowest stress applied (0.05 Pa). This is proof of a lack of structure in the continuous phase. Emulsions with very poor microstructure are usually kinetically unstable due to a creaming process. Concerning emulsions formulated with 0.2 wt.% and 0.3 wt.% of GG (Figure 3A), they exhibit very similar values of elastic modulus (G′) and viscous modulus (G″). However, the slope of the log-log plot of G′ versus angular frequency is higher for 0.2 wt.% than 0.3 wt.%, suggesting a higher elastic character of 0.3 wt.% GG emulsion. In addition, they show weak gel-like properties where G′ are higher than G″ values in all the frequency range studied and the occurrence of the so-called plateau zone, characterized by the plateau modulus, which may be related to the flocculation degree [25]. The values of plateau moduli are 4.5 and 6.3 Pa, respectively, which also demonstrate the higher elastic character of 0.3 wt.% GG emulsion.

On the other hand, emulsions with different concentrations of APXG present different behaviors (Figure 3B). Thus, a cross-over point between G′ and G″ is shown for the emulsion containing 0.1 wt.% APXG. This point indicates the end of the plateau zone and the onset of the transition zone of the relaxation spectrum. By contrast, 0.2 wt.% and 0.3 wt.% APXG emulsions present G′ higher than G″ in all the frequency range studied. Hence, a change of structuration (fluid-like to gel-like structure) is observed from 0.1 wt.% to 0.2 wt.% APXG. Furthermore, an increase in APXG concentration provokes an increase in G′ and G″ values for emulsions developed, as expected. Both facts are normally related to the formation of a highly structured continuous phase, but they also could be due to the formation of a flocculated droplet network [31].

**Figure 4:**
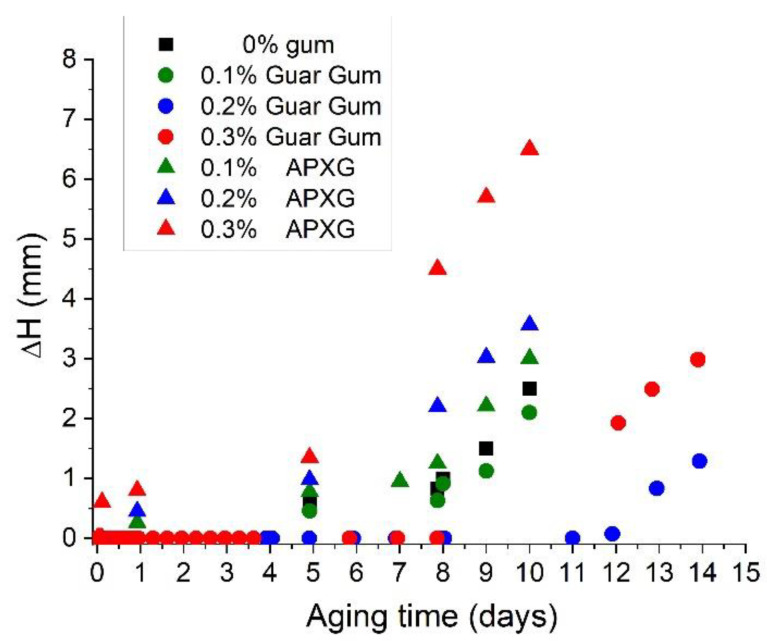
Height of the serum layer with aging time for linseed oil emulsions as a function of gum type and gum concentration.

The analysis of the multiple light scattering technique raw data shows that the main destabilization mechanism for all emulsions was the creaming process. The height of the serum layer with aging time is observed in Figure 4 for the emulsions developed. All the emulsions showed a creaming process with aging time but in different grades and with different delay times. Interestingly, emulsions formulated using APXG showed shorter delay times than their counterpart emulsions with GG. This is eye-catching since emulsions with APXG presented higher viscosities, which are normally required to reduce creaming. This fact points out that the increase in viscosity values and viscoelastic properties concerning APXG concentration is due to depletion flocculation, which depends on the molecular characteristics of the polysaccharide molecules [32]. This depletion flocculation induces a faster creaming process for emulsions containing APXG. It is important to note that the creaming induced by depletion flocculation in APXG systems is even faster than the emulsion without gum. In conclusion, emulsions formulated with GG showed longer physical stabilities. Nevertheless, the GG concentration above 0.2 wt.% provoked a reduction of physical stability, which indicates the occurrence of a depletion flocculation mechanism from this concentration.

### 3.3. Encapsulation of B-Carotene into Linseed Oil Emulsions

**Figure 5:**
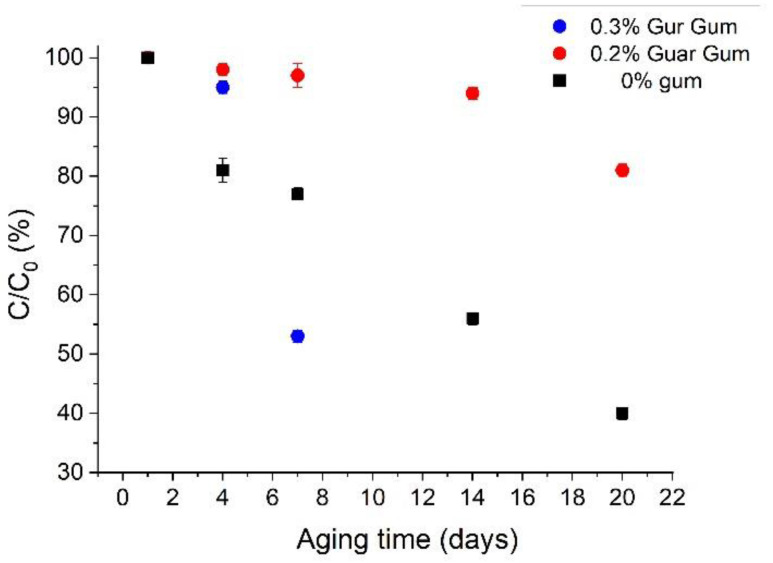
Influence of aging time on the chemical degradation of β-carotene encapsulated within the emulsions as a function of guar gum concentration and the reference sample (pure linseed oil) measured using a solvent extraction and spectroscopy method.

It is well-known that β-carotene is very sensitive to oxidation, heat, and light [33]. Hence, the influence of aging time on the relative β-carotene concentration remaining encapsulated in the oil droplets of the emulsions developed was studied (Figure 5). It is important to note that the solvent is taken as the reference sample (just the solvent without emulsification, i.e., pure linseed oil). In addition, the results for emulsions containing APXG and without gum are not shown because there was a precipitation of β-carotene a few days after preparation.

The encapsulation efficiency (*EE*) was calculated from the difference between the theoretical amount of β-carotene (*TC*) and the total amount of β-carotene available in the emulsion (*RC*).

(4) $$EE=\frac{TC-RC}{TC}\times 100$$

The calculated *EE* was 87.2%. This *EE* is similar to others obtained with orange essential oil [34] and sweet fennel oil [4]. Figure 5 shows a marked decrease in the relative β-carotene concentration with aging time for the reference sample (pure solvent) and for 0.3 wt.% GG emulsion. However, a slight decrease in the relative β-carotene concentration is clearly observed for 0.2 wt.% GG emulsion. Thus, the relative β-carotene concentration in the reference sample was about 40% in 21 days, whereas in the emulsion containing 0.2 wt.%, GG was even above 80% in the same period. This fact demonstrates that a sufficiently stable emulsion provides the needed protection to avoid the chemical degradation of β-carotene. Furthermore, this degradation is lower than in other emulsion-based delivery systems [35]. Interestingly, the emulsion containing 0.3 wt.% GG that displayed poor physical stability showed higher degradation than even the reference sample, which suggests the chemical degradation of β-carotene may be accelerated by the occurrence of the rest of the components of these emulsions [4,5].

**Figure 6:**
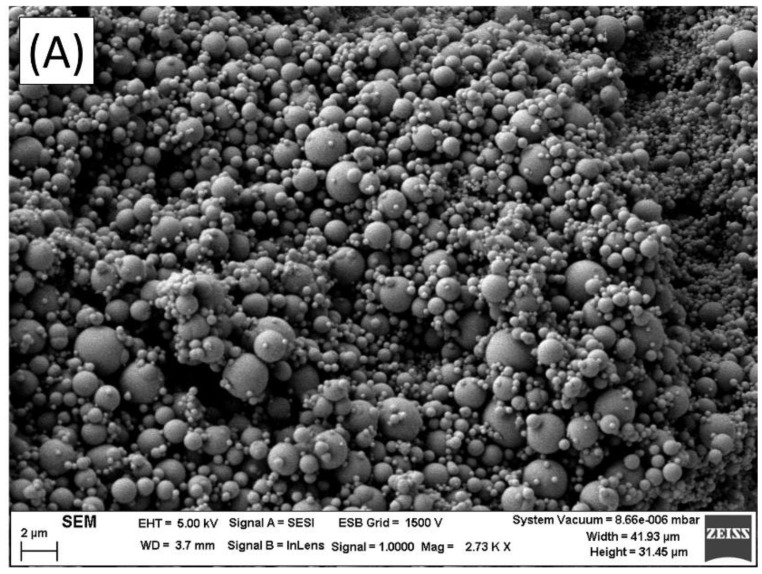

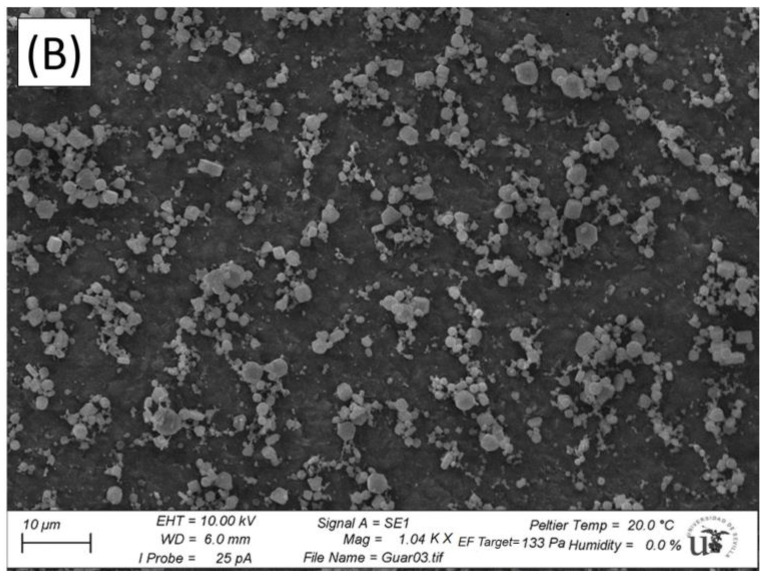
(**A**) Microstructure formed by linseed oil emulsion formulated with 0.3 wt.% of APXG observed by SEM. (**B**) Microstructure formed by linseed oil emulsion formulated with 0.3 wt.% of GG observed by SEM.

Figure 6A,B show the microstructure formed by 0.3 wt.% of APXG emulsion and 0.3 wt.% of GG emulsion, respectively. While extensive flocculation in all the emulsion is observed in Figure 6A (APXG), separate flocs are shown in Figure 6B (GG). This seems to prove that the extensive flocculation of APXG emulsions is caused by the fact that they are not stable with aging time. This was previously suggested by the plateau moduli values, related to the flocculation grade. In addition, the higher viscoelastic properties of APXG emulsions seem to be related to droplet aggregation. Moreover, the use of these gums did not provoke the occurrence of a strong 3D network observed in other emulsions [36]. Hence, not only GG but also APXG have a role as thickeners, enhancing the viscosity of the systems but not forming layers or networks.

## 4. Conclusions

The use of stable nanoemulsions as delivery vehicles is a strategy to protect lipophilic active ingredients with a high tendency to chemical degradation. We propose the use of two different types of thickeners (APXG and GG) at different concentrations in order to reach the required physical stability.

In this study, nanometric scale emulsions were obtained using the microfluidization technique. In all the samples studied, monomodal and narrow droplet size distributions (DSD) without recoalescence were observed, suggesting the effective role of the Appyclean 6552 as an emulsifier. The analysis of the processing variables (pressure and number of passes) revealed that the emulsion processed at 15,000 psi and two passes obtained the best results concerning DSD. Emulsions formulated with APXG or GG presented shear-thinning behavior with a trend to reach zero-shear viscosity at lower shear rates tested. However, it is important to note that the APXG provided emulsions exhibiting higher viscosities with a lower flow index and higher viscoelastic properties. These facts point out that APXG either generates more structured continuous phases or induces highly flocculated systems. The same finding was supported by the viscoelastic properties of emulsions. The increase in APXG concentration provokes a change in viscoelastic behavior from fluid-like to gel-like. On the other hand, emulsions formulated with GG show the presence of a plateau window in the mechanical spectra, characterized by plateau modulus, which is higher for 0.3 wt.% GG emulsion. This brings to light that depletion flocculation may be induced above a certain concentration depending on the nature of the polysaccharide. In this sense, multiple light scattering results demonstrated a higher flocculation grade for emulsions containing APXG through the occurrence of a more marked creaming, as supported by electronic microscopy. In the same line, emulsion with 0.3 wt.% GG presented a higher flocculation grade than 0.2 wt.% GG. The β-carotene encapsulation study highlights that the physical stability strongly determines the encapsulation efficiency. Thus, emulsions possessing poor physical stability showed very low encapsulation efficiency. Hence, this work emphasizes the importance of the adequate choice of a polysaccharide and its concentration to obtain nanoemulsions with appropriate stability for active ingredient-delivery applications.

## Figures and Tables

**Table 1:** Sauter diameter, volumetric diameter, *D*_10_, *D*_50_, *D*_90,_ and span values for linseed oil nanoemulsions as a function of homogenization pressure and number of passes using the Microfluidizer.

Sample	*D*_3,2_ (μm)	*D*_4,3_ (μm)	*D*_10_ (μm)	*D*_50_ (μm)	*D*_90_ (μm)	Span
Pre-emulsion	2.333	3.698	1.056	3.531	6.495	1.540
15,000 psi/1 pass	0.295	0.334	0.194	0.315	0.504	0.984
15,000 psi/2 passes	0.239	0.267	0.164	0.245	0.389	0.965
20,000 psi/1 pass	0.299	0.343	0.195	0.321	0.525	1.029
20,000 psi/2 passes	0.238	0.265	0.158	0.233	0.384	0.954
25,000 psi/1 pass	0.255	0.288	0.171	0.265	0.440	1.011
25,000 psi/2 passes	0.258	0.296	0.171	0.268	0.465	1.097

**Table 2:** Fitting parameter for linseed oil emulsions as a function of gum type and gum concentration.

Gum Concentration (wt.%)	η_0_ (mPa·s)	η_∞_ (mPa·s)	k	n
0 wt.% gum	Ostwald de Waele model			
0.1 wt.% Guar Gum	0.16	0	0.55	0.52
0.2 wt.% Guar Gum	651	0.05	3150	0.2
0.3 wt.% Guar Gum	1105	0.1	4236	0.2
0.1 wt.% APXG	221	0.02	1554	0.21
0.2 wt.% APXG	7392	0.02	10200	0.2
0.3 wt.% APXG	10748	0.05	3529	0.15

## Data Availability

Not applicable.
